# Proceedings of the Hahnemann Club of Toronto

**Published:** 1888-03

**Authors:** J. D. Tyrrell

**Affiliations:** Secretary-Treasurer


### PROCEEDINGS OF THE HAHNEMANN CLUB OF TORONTO.

Regular meeting of the Hahnemann Club opened at half-past eight P. M., Wednesday, February 1st, the President, Dr. Hall, in the chair. After reading of the minutes of last meeting, the news of the death of Dr. Adolph Lippe was received in sadness by the Club, and it was therefore

*Resolved,* That in the death of Dr. Lippe each member feels that he has lost a teacher and (in common with *all Hahnemannians*) a personal friend; that our tenderest sympathy is extended to his family in this, their bereavement, and that this tribute to his memory be placed on our records. The following papers on Post-partum Hemorrhage were read: * * * * * Remedies for consideration next meeting are: Cinnamomum, Phos., Puls., Sabina, Secale, and Ustilago.

J. D. Tyrrell, *Secretary-Treasurer.*

